# Profiling the autoantibody repertoire reveals autoantibodies associated with mild cognitive impairment and dementia

**DOI:** 10.3389/fneur.2023.1256745

**Published:** 2023-11-30

**Authors:** Hanan Ehtewish, Areej Mesleh, Georgios Ponirakis, Katie Lennard, Hanadi Al Hamad, Mani Chandran, Aijaz Parray, Houari Abdesselem, Patrick Wijten, Julie Decock, Nehad M. Alajez, Marwan Ramadan, Shafi Khan, Raheem Ayadathil, Ahmed Own, Ahmed Elsotouhy, Omar Albagha, Abdelilah Arredouani, Jonathan M. Blackburn, Rayaz A. Malik, Omar M. A. El-Agnaf

**Affiliations:** ^1^College of Health and Life Sciences (CHLS), Hamad Bin Khalifa University (HBKU), Qatar Foundation (QF), Doha, Qatar; ^2^Neurological Disorders Research Center, Qatar Biomedical Research Institute (QBRI), Hamad Bin Khalifa University (HBKU), Qatar Foundation (QF), Doha, Qatar; ^3^Department of Medicine, Weill Cornell Medicine-Qatar, Qatar Foundation (QF), Doha, Qatar; ^4^Sengenics Corporation, Level M, Plaza Zurich, Damansara Heights, Kuala Lumpur, Malaysia; ^5^Geriatric and Memory Clinic, Rumailah Hospital, Hamad Medical Corporation (HMC), Doha, Qatar; ^6^The Neuroscience Institute, Academic Health System, Hamad Medical Corporation (HMC), Doha, Qatar; ^7^Proteomics Core Facility, Qatar Biomedical Research Institute (QBRI), Hamad Bin Khalifa University (HBKU), Qatar Foundation (QF), Doha, Qatar; ^8^Diabetes Research Center, Qatar Biomedical Research Institute (QBRI), Hamad Bin Khalifa University (HBKU), Qatar Foundation (QF), Doha, Qatar; ^9^Translational Cancer and Immunity Center, Qatar Biomedical Research Institute (QBRI), Hamad Bin Khalifa University (HBKU), Qatar Foundation (QF), Doha, Qatar; ^10^Department of Neuroradiology, Hamad General Hospital, Hamad Medical Corporation, Doha, Qatar; ^11^Department of Clinical Radiology, Weill Cornell Medicine-Qatar, Qatar Foundation, Doha, Qatar; ^12^Department of Integrative Biomedical Sciences, Faculty of Health Sciences, University of Cape Town, Cape Town, South Africa; ^13^Faculty of Health Sciences, Institute of Infectious Disease and Molecular Medicine, University of Cape Town, Cape Town, South Africa

**Keywords:** neurodegeneration, dementia, MCI, blood, autoantibodies, mechanism, pathway

## Abstract

**Background:**

Dementia is a debilitating neurological disease affecting millions of people worldwide. The exact mechanisms underlying the initiation and progression of the disease remain to be fully defined. There is an increasing body of evidence for the role of immune dysregulation in the pathogenesis of dementia, where blood-borne autoimmune antibodies have been studied as potential markers associated with pathological mechanisms of dementia.

**Methods:**

This study included plasma from 50 cognitively normal individuals, 55 subjects with MCI (mild cognitive impairment), and 22 subjects with dementia. Autoantibody profiling for more than 1,600 antigens was performed using a high throughput microarray platform to identify differentially expressed autoantibodies in MCI and dementia.

**Results:**

The differential expression analysis identified 33 significantly altered autoantibodies in the plasma of patients with dementia compared to cognitively normal subjects, and 38 significantly altered autoantibodies in the plasma of patients with dementia compared to subjects with MCI. And 20 proteins had significantly altered autoantibody responses in MCI compared to cognitively normal individuals. Five autoantibodies were commonly dysregulated in both dementia and MCI, including anti-CAMK2A, CKS1B, ETS2, MAP4, and NUDT2. Plasma levels of anti-ODF3, E6, S100P, and ARHGDIG correlated negatively with the cognitive performance scores (MoCA) (*r*^2^ –0.56 to −0.42, value of *p* < 0.001). Additionally, several proteins targeted by autoantibodies dysregulated in dementia were significantly enriched in the neurotrophin signaling pathway, axon guidance, cholinergic synapse, long-term potentiation, apoptosis, glycolysis and gluconeogenesis.

**Conclusion:**

We have shown multiple dysregulated autoantibodies in the plasma of subjects with MCI and dementia. The corresponding proteins for these autoantibodies are involved in neurodegenerative pathways, suggesting a potential impact of autoimmunity on the etiology of dementia and the possible benefit for future therapeutic approaches. Further investigations are warranted to validate our findings.

## 1 Introduction

Dementia is a complex neurodegenerative syndrome that affects millions of people worldwide. Pathologically characterised by disease-specific protein aggregation in the brain. The pathogenic role of abnormal protein deposition in dementia is established (1, 2); however, the exact mechanisms for the initiation and progression of neurodegeneration remain unclear. There is increasing evidence for the role of immune dysregulation in the pathogenesis of dementia. Genome-wide association studies have demonstrated common genetic variations in the immune system processes associated with neurodegeneration in Alzheimer’s disease (AD), Parkinson’s disease dementia, and frontotemporal dementia (FTD) (3–5). Indeed, the role of autoimmune mechanisms is increasingly recognized in the pathophysiology of dementia (6–8). Autoantibodies are self-antigens recognizing antibodies, produced during B cell development in healthy individuals and play a role in immune tolerance and homeostasis (9). However, through various genetic and environmental processes, the ability to distinguish “self” from “non-self” antigens deteriorates, enabling autoantibodies to initiate and maintain the inflammatory cascade responsible for tissue injury (10, 11).

Autoantibodies have been detected in the blood and cerebrospinal fluid of patients with different forms of dementia, including autoimmune dementia and neurodegenerative dementias such as AD, vascular dementia (VD), FTD, and Lewy body dementia (DLB) (12–16). Autoimmune dementia is a progressive cognitive impairment characterised by early onset, atypical clinical presentation and rapid progression associated with neural antibodies, inflammation in cerebrospinal fluid, brain changes in MRI atypical for neurodegenerative disease, and a good response to immunotherapy (17). Various neural autoantibodies were frequently detected in individuals experiencing progressive cognitive decline. These autoantibodies often target cell surface proteins such as the N-methyl-D-aspartate receptor (NMDAR), gamma-aminobutyric acid B receptor (GABABR), alpha-amino-3-hydroxy-5-methyl-4-isoxazolepropionic acid receptor (AMPAR), leucine-rich glioma inactivated protein 1 (LGI1), and dipeptidyl-peptidase protein-like 6 (DPPX) (18, 19). However, there is an overlap in the neural autoantibodies profile in autoimmune dementia with neurodegenerative dementias such as FTD and DLB (20).

One of the most well-known forms of dementia is AD, which is characterized by the buildup of amyloid plaques and neurofibrillary tangles in the brain (21). Autoantibodies targeting β-amyloid, tau, neurotransmitters and microglia have been reported in patients with AD (6, 11, 22, 23). Autoantibodies targeting Aβ are decreased in the AD patients (24, 25), and are deemed to play a protective role against Aβ toxicity (26, 27). Additionally, patients with AD have been shown to have increased levels of autoantibodies against glutamate (28), oxidized low-density lipoproteins (29), glial markers such as GFAP and S100b (30, 31), and receptors for advanced glycosylation end products (32) in the serum or cerebrospinal fluid. Autoantibodies have been detected in patients with MCI (33, 34), suggesting the potential role of autoimmunity on disease initiation. Although autoantibodies against molecules related to different forms of dementia pathology have been discovered, further research is required to assess the potential of detecting autoantibodies to serve as diagnostic/prognostic biomarkers and in the development of effective immunotherapies for dementia (26).

The use of protein microarrays for autoantibody profiling has been suggested as a potential method to screen for novel autoantibodies to aid in the diagnosis and monitoring of MCI and dementia. This pilot study utilised a functional protein microarray platform developed by Sengenics patented KREX technology to comprehensively profile more than 1,600 autoantibodies in the blood of patients with dementia, MCI and cognitively normal healthy controls. KERX technology is based on the use of the biotin carboxyl carrier protein (BCCP) to ensure that only correctly folded, full-length proteins are immobilised on the arrays. That allows maximum epitope binding to discover the plasma autoantibody. The quantitative signal measured on the arrays for each autoantibody-autoantigen pair is directly proportional to the autoantibody concentration in the blood.

## 2 Materials and methods

### 2.1 Participants and plasma collection

Plasma samples of 127 participants [50 cognitively normal controls, 55 subjects with MCI, and 22 subjects with dementia including those with AD (*n* = 3), VD (*n* = 5), and mixed dementia (*n* = 14)]. The participants were recruited over 3 years (2019–2021) as part of a prospective study in the Geriatric and Memory clinic in Rumailah Hospital (Doha, Qatar). Additional control samples were collected from the Qatar Biobank (Doha, Qatar). Blood samples were collected in EDTA tubes and centrifuged at 1500× *g* for 15 min at 4°C and the plasma was isolated, collected and stored at −80°C. This study was approved by the Qatar Biomedical Research Institute (2019-013), Hamad Medical Corporation (RP14494/14), and Weill Cornell Medicine-Qatar (15-00019), Doha, Qatar, in accordance with applicable guidelines and regulations. Written informed consent was obtained from all participants.

### 2.2 Diagnostic procedures

All subjects underwent standardised investigation at a memory disorder unit by geriatricians, geriatric psychiatrists and neurologists, including clinical assessment and history of illness, caregiver interviews, clinical neurological examination, neuropsychological evaluation, functional history of essential daily living activities, neuroimaging, and laboratory assessments for ruling out other causes of cognitive dysfunction. The diagnosis of MCI and dementia was based on the International Classification of Diseases, Tenth Revision (ICD-10) criteria. AD diagnosis was based on MRI typical features of AD based on a blinded assessment by neuroradiologists to identify volume loss of hippocampi, entorhinal cortex, and amygdala according to the criteria of Dubois et al. (35). The diagnosis of VD was based on the National Institute of Neurological Disorders and Stroke-Association Internationale pour la Recherche et l’Enseignement en Neurosciences (NINDS-AIREN) criteria (36), which includes the presence of multiple large vessel infarcts or a single strategically placed infarct (angular gyrus, thalamus, basal forebrain, or posterior (PCA) or anterior cerebral artery (ACA) territories), or multiple lacunes in the basal ganglia and white matter, or extensive periventricular white matter lesions, or combinations thereof. The mixed dementia diagnosis was based on the presence of AD features and significant vascular changes.

### 2.3 Cognitive function assessment

Cognitive function was assessed using the Montreal Cognitive Assessment (MoCA) test. MoCA assesses seven cognitive domains, including visuospatial/executive, naming, memory, attention, language, abstraction and delayed recall giving a total score of 30. A score of ≤26 indicates cognitive impairment. A point was added for individuals who had formal education ≤6th grade. Cognitive symptom duration was estimated from the clinical history obtained from relatives and participants.

### 2.4 Autoantibody measurement

Samples were analyzed for more than 1,600 autoantibodies using Immunome protein arrays (Sengenics, Singapore), developed using (KREX) technology to provide a high-throughput immunoassay based on correctly folded, full length and functional recombinant human proteins. The Immunome arrays contain antigens enriched for kinases, signaling molecules, cytokines, interleukins, and chemokines, as well as known autoimmune- and cancer antigens. Additionally, using Stringdb [STRING: functional protein association networks (string-db.org)] searching with the list of 1,609 proteins, 201 (12%) are known proteins matching cognitive impairment. Samples were diluted in Serum Albumin Buffer (SAB) at optimized dilution (1:200) and microarray slides were prepared in four-well plates/slide. Samples, including controls, were randomized and applied to the microarray slides for 2 h, and samples’ IgGs were then detected by secondary Cy3-labeled IgG antibodies. Slides were scanned at a fixed gain setting using the Agilent G4600AD fluorescence microarray scanner at 10 μm resolution, generating a 16-bit TIFF file.

### 2.5 Data retrieval, normalization and pre-processing

A GAL (GenePix Array List) file containing information regarding the location and identity of all probed spots was used to aid with image analysis. Automatic extraction and quantification of each spot were performed using GenePix Pro software (Molecular Devices). For each spot, the background signal intensity was calculated using a circular region with three times the diameter of the spot, centred on the spot (i.e., the local background). The net intensity was calculated by subtracting the background from the foreground to reduce any additive effects the background noise might have on the foreground values. Biotinylated human IgG (detected by fluorescently labeled secondary antibody) and biotinylated human anti-IgG (detected only when plasma or serum is added to the slide) were used as positive controls to assess assay integrity. Sengenics autoantibody quantitation limit is in the pg/mL range (million-fold serum dilution). By extrapolating of the linear range to the lower limit and assuming a noise threshold of two standard deviations (SD) of the background, the detection limit was estimated to be ~1:1,000,000 serum dilution, corresponding to an autoantibody titer of ~190 pg/mL. The data obtained were processed through filtering and normalization. The process involved subtracting the median background pixel intensity (RFU) from the median foreground pixel intensity (RFU) for each antigen to obtain the median net intensity per spot (RFU). Next, each antigen’s coefficient of variation (CVs) was calculated based on the quadruplicate technical replica spots on a given array. Antigens with CV values above 20% were flagged, and their outlier spots were removed as long as at least two valid values remained. The net intensity values for each antigen in a particular sample were calculated by obtaining the mean of the net intensity values for technical replica spots on that array. Finally, the data were normalized across replica arrays using the Cy3-BSA controls. Furthermore, the generated dataset underwent negative control filtration (NCF), a technique used to remove non-specific autoantibody signals. Signals that exhibited a high correlation with the negative controls were excluded from downstream analysis. Before conducting further analyses on any dataset, data normalization is employed to eliminate any systematic experimental variations within the dataset. In this study, we applied the Loess normalization approach described by Liu et al. (37). The Loess normalization method fits a nonlinear Loess signal, assigning equal weight to all data points. It typically addresses intensity-dependent bias within the representation of the data in the heatmap of net intensity. This method comprehensively assesses the entire dataset to estimate and mitigate any potential systematic variations or batch effects. To execute this, we utilised the “normalizeCyclicLoess” function, as introduced by Ballman et al. (38). Additionally, to address batch effects caused by the two different runs performed in 2021 and 2022, Combat correction was applied using the empirical Bayesian approach (39). A t-SNE plot of the data before and after normalization is shown in Supplementary Figure S1.

### 2.6 Statistical analysis

All statistical analysis was performed using R version 4.2.2 (2022-10-31). Continuous clinical variables were reported as the mean ± standard deviation. Differential expression analysis between the groups (dementia versus control and dementia versus MCI) was conducted using the limma (Linear Models for Microarray Data) package in R, adjusting for age, gender, and diabetes status. The significance threshold of a value of *p* < 0.05 was used. The correlation between MoCA score of participants with dementia and MCI and the plasma autoantibodies levels was computed by Spearman’s correlation coefficients (*r*^2^) using cor.test() function in the R. All plots were generated using R, the heatmap() function from the ComplexHeatmap package was used to generate a heatmap of the top dysregulated plasma autoantibodies, and a volcano plot was generated using the ggplot() function, and the correlation plots ggscatter() function in R was utilized.

### 2.7 Protein functional enrichment analysis

To identify overrepresented cellular components (CCs), molecular functions (MFs), biological processes (BPs), and KEGG pathways, we performed protein enrichment analysis using ShinyGO (version 0.77)^1^ on the corresponding proteins of the differentially expressed autoantibodies. The Fisher exact test (FET) was used to test all enriched terms, with an adjusted FDR value of *p* < 0.05.

### 2.8 Sequence identity analysis

To assess cross-reactivity among proteins that express similar antigen epitopes and are highly correlated, we checked the correlation of the differentially expressed autoantibodies. The corresponding proteins of the highly correlated autoantibodies (*r*^2^ > 0.7) were then aligned with the highly correlated proteins using the Uniport alignment tool.

## 3 Results

### 3.1 Demographic and clinical characteristics

**Figure 1:**
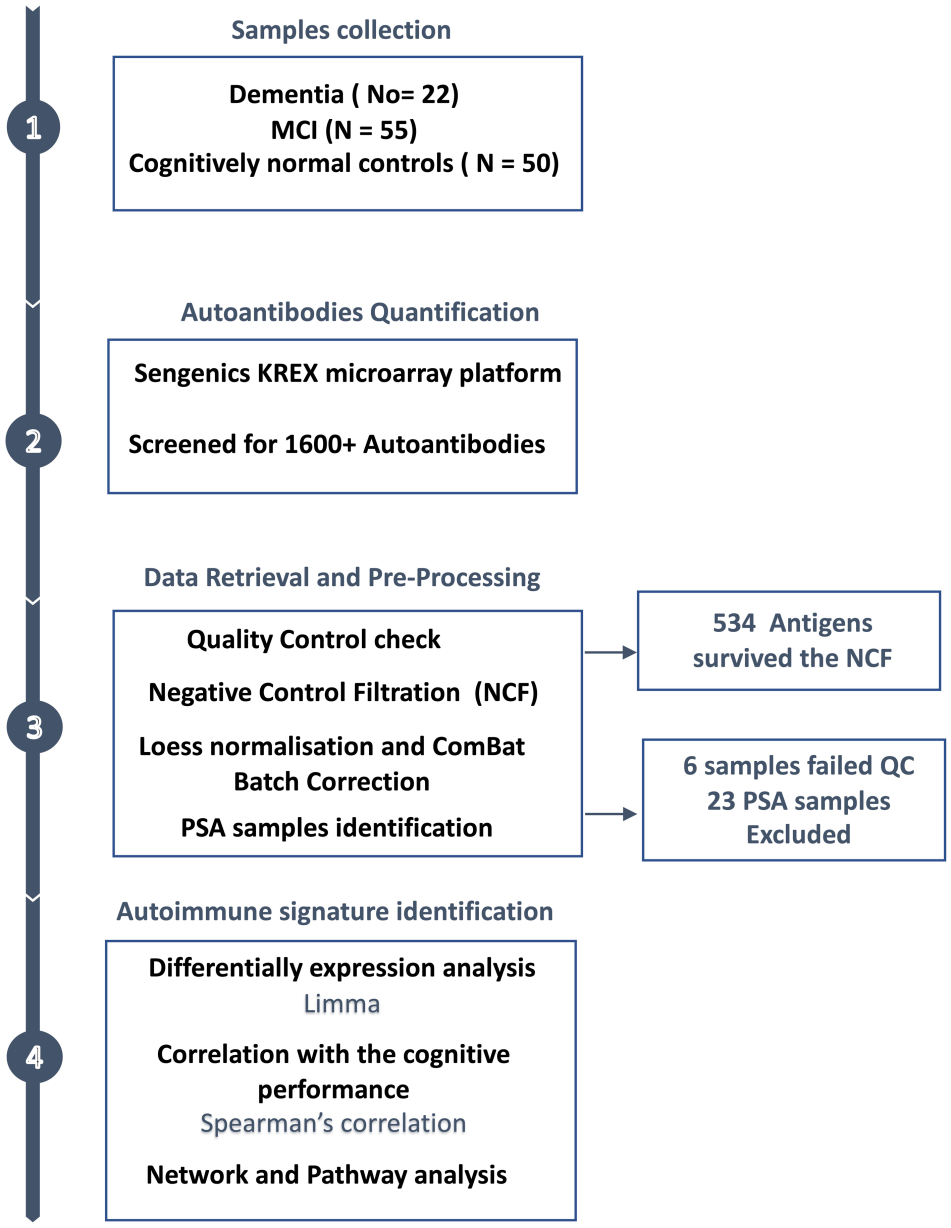
Study design overview. Plasma samples were collected from subjects with dementia (*n* = 22), MCI (*n* = 55) and cognitively normal controls (*n* = 50), along with the participants’ clinical and demographic information. The plasma autoantibodies were measured using KREX technology immunoassay.

An overview of the study design is illustrated in Figure 1. Plasma from 127 subjects, including 22 with dementia, 55 with MCI, and 50 cognitively normal individuals were applied to the KREX Immunome™ Discovery microarray (Sengenics). On visualising the screened samples and autoantibodies on a heatmap, we identified a pattern of polyspecific antibody (PSA) reactivity in 23 samples (Supplementary Figure S2). This pattern occurs when the antibodies retain low-affinity characteristics with a spectrum of antigens, including self-antigens. This phenomenon occurs with some types of infection, recent vaccination and autoimmune conditions (40). All samples that displayed PSA features were removed, in addition to another six samples that failed the quality check. This made totals 98 samples that were subjected to downstream analysis, including 38 cognitively normal controls, 42 subjects with MCI, and 18 subjects with dementia. The participants were 66 ± 7, 70 ± 8, and 75 ± 5 years old, respectively. The majority of dementia subjects were male (66%); however, in the controls and MCI groups, the male: female ratio was approximately equal (50%). The MoCA score differed significantly (adjusted value of *p* < 0.05) between the groups, with an average MoCA score of 11.6 ± 6.6 in the group with dementia, 22.9 ± 6.6 in those with MCI and 28.9 ± 1.4 in cognitively normal subjects. The clinical and demographic information of the participants is summarised in Table 1.

**Table 1:** Main clinical and demographic characteristics of the participants.

	Control	MCI	Dementia
Sample size	*N* = 50	*N* = 55	*N* = 22
Age in years (mean ± SD)	66 ± 7	70 ± 8	75 ± 5
Gender (F:M)	18:32	25:30	8:14
MoCA score (mean ± SD)	28.9 ± 1.4	22.9 ± 6.6	11.6 ± 6.6

All data are presented as mean (SD). MoCA, Montreal Cognitive Assessment; MCI, Mild cognitive impairment; SD, Standard deviation.

### 3.2 Autoantibodies profiling and enrichment functional pathway analysis in dementia and MCI

#### 3.2.1 Autoimmunomic signatures of dementia

**Figure 2:**
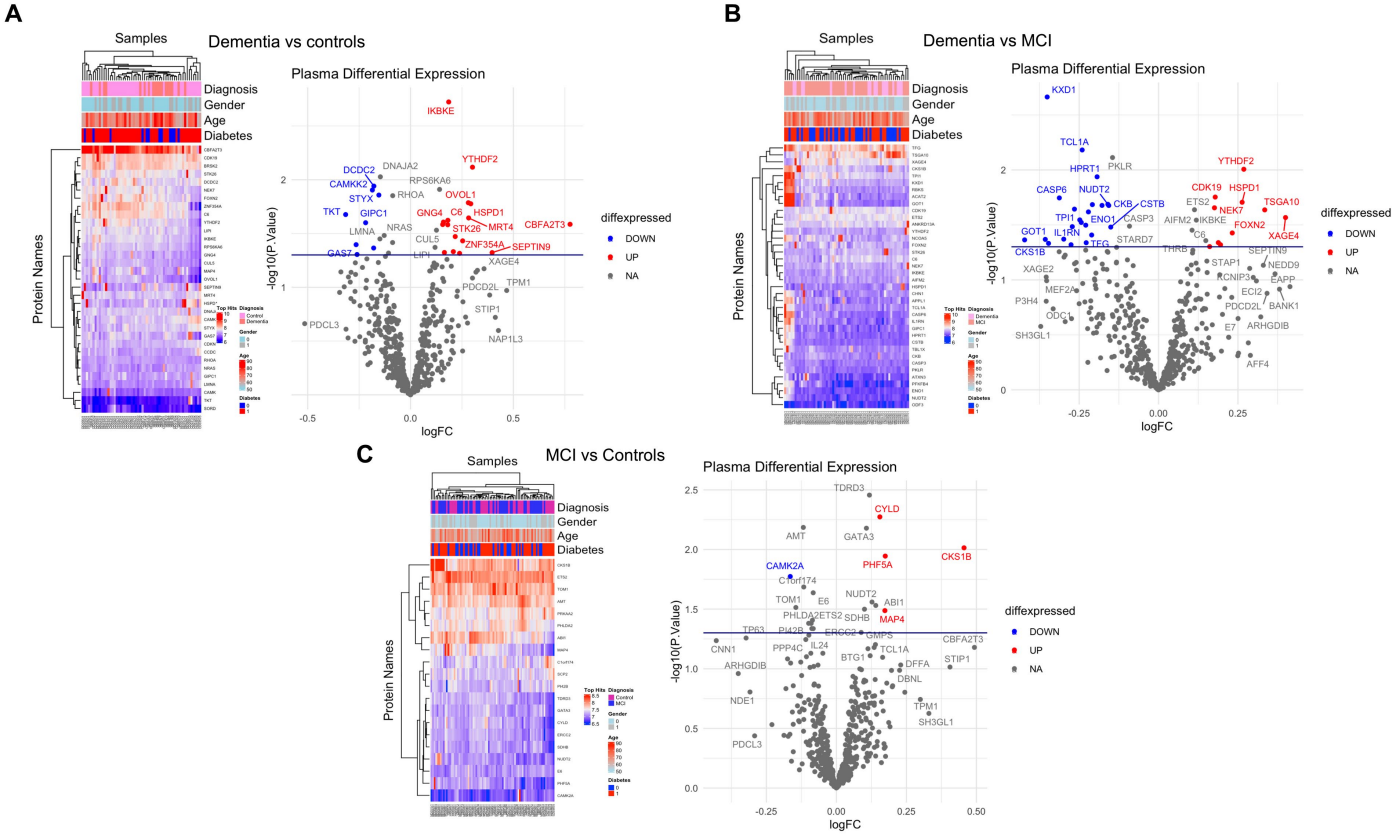
Differential expression of plasma autoantibodies **(A)** Dementia vs. cognitively normal controls; **(B)** Dementia vs. MCI; **(C)** MCI vs. cognitively normal controls. Supervised heatmaps across the control and cases using the top significantly altered autoantibodies in the dataset (*p* < 0.05); volcano plot displaying the log2 fold change (*x*-axis) against the limma-derived –log10 statistical value of *p* (*y*-axis) for all autoantibodies differentially expressed in dementia and MCI cases. Proteins with significantly decreased levels in dementia and MCI (*p* < 0.05) are shown in blue, while the proteins with significantly increased levels are shown in red. Select proteins are labeled.

**Figure 3:**
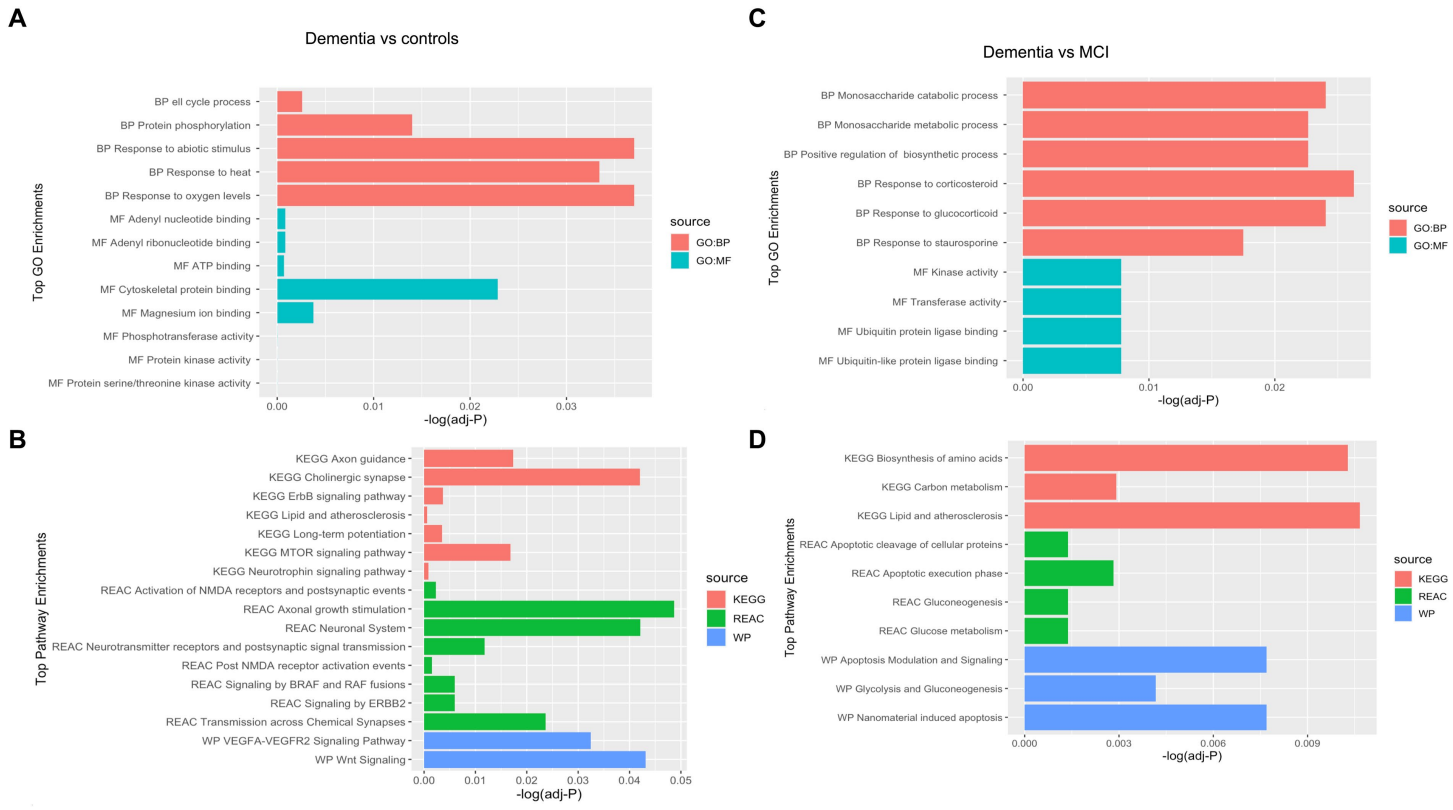
Gene Ontology (GO) terms associated with the Corresponding proteins to the significantly altered autoantibodies in dementia in the domains of biological process, molecular function, cellular component and KEGG and REAC pathways are shown. **(A)** Top GO enrichment terms in Dementia vs controls, **(B)** Top Pathways enrichment in Dementia vs controls, **(C)** Top GO enrichment terms in Dementia vs MCI, **(D)** Top Pathways enrichment in Dementia vs MCI.

Of 534 autoantibodies that passed the NCF and quality control measures, differential expression analysis by Limma identified 33 proteins that were recognized by autoantibodies in the plasma of patients with dementia compared to cognitively normal controls (*p* < 0.05). The significant autoantibodies’ expression levels are illustrated on the heatmap (Figure 2A) and for more details (Supplementary Table S1). In patients with dementia, 14 autoantibodies were upregulated, while 19 autoantibodies were downregulated. Seven differentially expressed autoantibodies were recognised as cognitive impairment-related, including HSPD1, CDK19, TKT, BRSK2, NRAS, LMNA, and CAMK2A. Enrichment annotation analysis showed that antigens targeted by autoantibodies dysregulated in dementia were significantly enriched in GO terms for catalytic or binding activity. Moreover, multiple autoantibodies corresponding to antigens overrepresented in the molecular function GO terms were mainly for cell cycle and protein phosphorylation processes (Figure 3A). Several autoantibodies against proteins enriched in neuro-related pathways were identified based on KEGG and Reactome databases. These pathways included neurotrophin signaling, long-term potentiation, axon guidance, cholinergic synapses, neurotransmitter receptors, postsynaptic signal transmission, and chemical synapses (Figure 3B). These pathways commonly involved four corresponding proteins: RHOA, CAMK2A, RPS6KA6, and NRAS. Other differentially expressed autoantibody corresponding proteins were enriched in signaling pathways such as ErbB, VEGFA-VEGFR2 and Wnt signaling pathways; these are autoantibodies against CAMK2A, CDKN1A, NRAS, GIPC1, CUL5, and LMNA proteins. Additionally, neurodegenerative disease and amnestic disorder were among the disease alliance enriched terms and included MAP4 and RHOA proteins (FDR = 0.042).

#### 3.2.2 Identification of autoantibodies altered in advanced stage of cognitive dysfunction

Comparing the dementia group to MCI, the autoimmune analysis identified 38 autoantibodies to be differentially expressed in the plasma of dementia (Figure 2B; Supplementary Table 2). Twenty-four of these differentially expressed autoantibodies were downregulated, while 14 were upregulated in dementia. In addition, nine autoantibodies were commonly dysregulated in dementia compared to both cognitively normal controls and MCI. Out of the 201 proteins recognised by String network analysis as cognitive impairment related, 5 autoantibodies targeting HPRT1, CDK19, HSPD1, ATXN3 and CSTB proteins were altered in the plasma of dementia patients. Based on the GO enrichment analysis, several antigens targeted by autoantibodies dysregulated in dementia were significantly enriched in GO terms of catalysis; CASP6 and CASP3 are particularly involved in endopeptidase activity. Furthermore, in dementia many of the autoantibodies were altered in relation to biological processes GO terms, including regulation of metabolic and biosynthetic processes as well as cellular responses to staurosporine, glucocorticoids, nitrogen compounds, and cytokines (Figure 3C). The protein pathway analysis revealed that some of the proteins targeted by the autoantibodies were involved in apoptosis, lipid and atherosclerosis, glycolysis and gluconeogenesis pathways (Figure 3D) and included ENO1, TPI1, GOT1, STK26, CASP6, CASP3, APPL1, HSPD, and IKBKE. According to the disease alliance terms, ENO1, CASP6, HSPD1, and CASP3 are associated with Alzheimer’s disease. Moreover, CASP3, CASP6, as well as GOT1 and IL1RN, have been implicated in vascular dementia, middle cerebral artery infarction and transient cerebral ischemia (FDR = 0.041).

#### 3.2.3 MCI-related autoantibody profile

The autoantibody profiling analysis revealed that 20 proteins had significantly altered autoantibody responses in MCI compared to cognitively normal individuals with value of *p*s of 0.05; ten were upregulated, and ten were downregulated (Figure 2C; Supplementary Table S3). Five autoantibodies (CAMK2A, CKS1B, ETS2, MAP4, and NUDT2) were dysregulated in both dementia and MCI. Additionally, based on the String network analysis, the corresponding autoantibodies to 4 neuronal-related antigens were dysregulated in the plasma of MCI participants, including CAMK2A, AMT, ERCC2, and SDHB. The autoantibodies’ corresponding proteins in MCI were overrepresented in the nucleoplasm and involved in catalysis and binding activities, but no activated pathways were identified. MAP4 was upregulated in MCI and was revealed to be associated significantly with tauopathy and amnestic disorder terms in disease alliance (FDR = 0.021).

### 3.3 Association between autoantibody profile and cognitive performance

**Figure 4:**
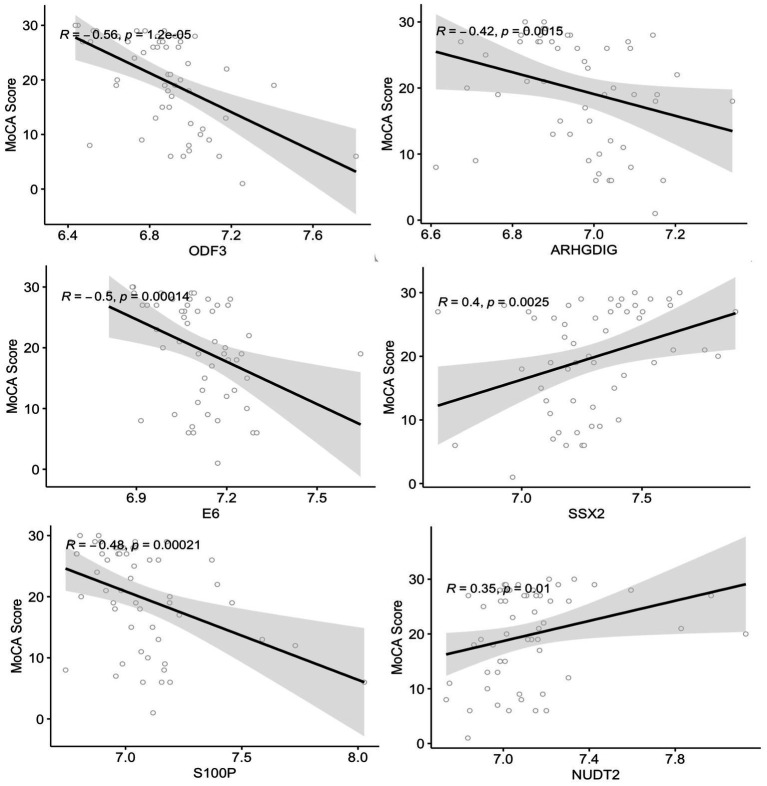
Correlation analysis between the plasma autoantibodies and the cognitive function score (MoCA). The top six correlations between autoantibodies and the MoCA score with a correlation coefficient >0.3 and <−0.4 (value of *p* < 0.05).

The correlation between plasma autoantibody levels and the degree of cognitive decline measured by MoCA scores in subjects with MCI and dementia was determined using Spearman’s correlation. The analysis revealed 33 proteins with significant correlations to MoCA scores (*r*^2^ ≥ 0.27 and ≤−0.27, value of *p* < 0.05) (Supplementary Table S4). There was a significant negative correlation between 20 autoantibodies and the MoCA score, of which ODF3, E6, S100P, and ARHGDIG, showed the strongest correlations with *r*^2^ between – 0.56 and – 0.42, value of *p* < 0.001, respectively (Figure 4). Two of the negatively correlated autoantibodies, ETS2 and ODF3, were dysregulated in dementia, and three were dysregulated in MCI. There were 13 positively correlated proteins, but only the SSX2 protein had an *r*^2^ of 0.40 (Figure 4). Of these significantly positively correlated autoantibodies, five were differentially expressed in dementia and included CL1A, CBFA2T3, PKLR, APPL1, and NUDT2, whilst two were dysregulated in MCI including ABI1 and NUDT2.

### 3.4 Cross-reactivity analysis to verify specific epitope recognition of identified autoantibodies

**Figure 5:**
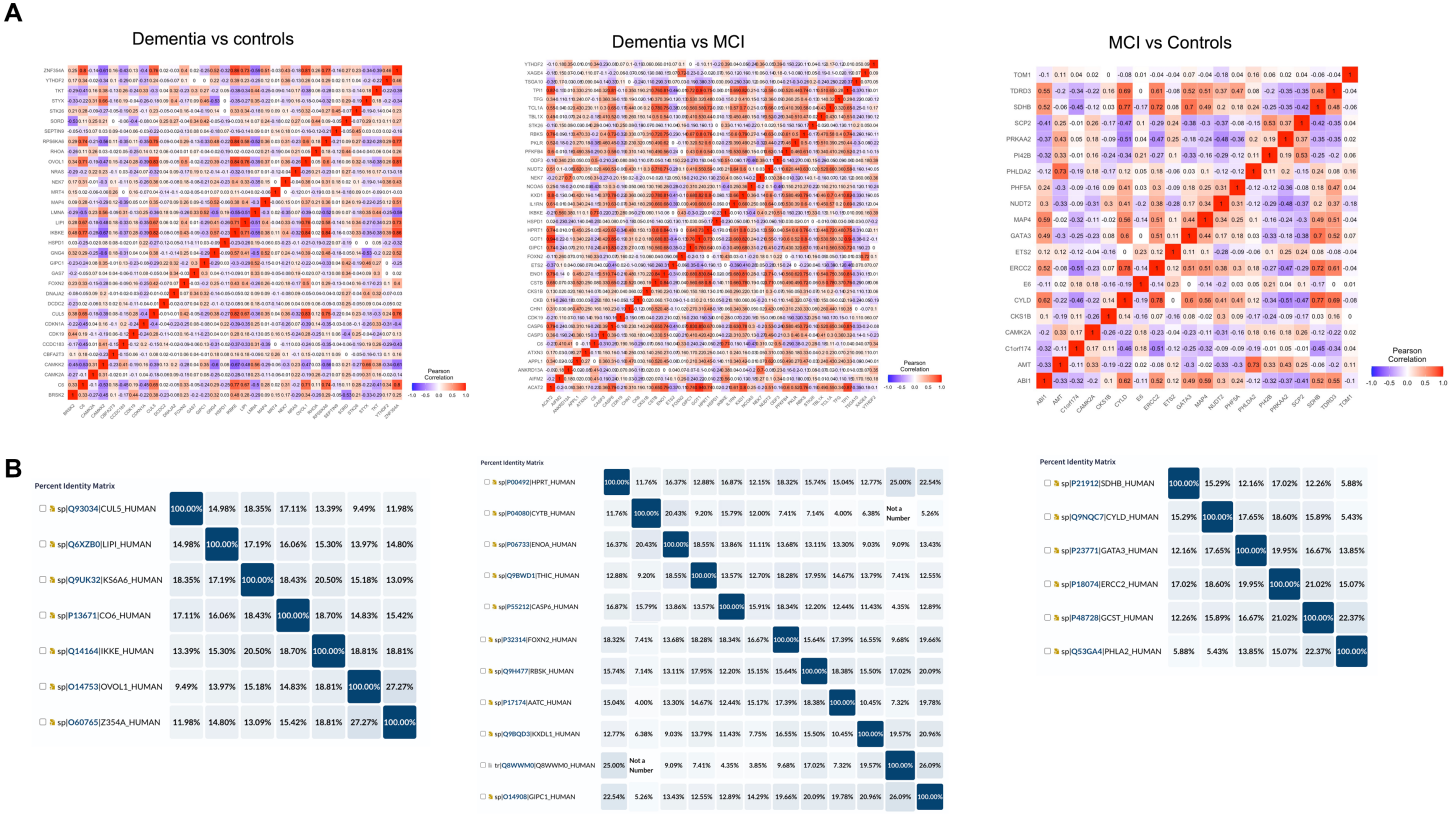
Cross-reactivity checks for the identified differential autoantibodies. **(A)** A correlation matrix of the differential autoantibodies using Pearson’s correlation, red = high and blue = low. **(B)** Identity matrix, generated from aligning the corresponding protein sequences of the highly correlated autoantibodies (*r*^2^ ≥ 0.7).

In order to determine cross-reactivity, a correlation analysis was performed on differentially expressed autoantibodies identified by Limma. Highly correlated autoantibodies (*r*^2^ > 0.7) were subjected to sequence alignment and identity analysis. The correlation matrix (Figure 5A) indicated seven correlated autoantibodies in dementia vs. cognitively normal controls, 12 in dementia vs. MCI, and 6 in MCI vs. controls. Alignment and identification analysis of those protein sequences revealed that all proteins were less than 28% identical. Furthermore, the fact that these proteins were not even isoforms suggested a low probability of cross-reactivity (Figure 5B). Therefore, the observed correlation may be attributed to biological or functional relevance rather than antibody cross-reactivity.

## 4 Discussion

The complexity of mechanisms underlying cognition, especially in neurodegenerative disorders with the involvement of multiple biological and environmental factors confers a major challenge for the extensive neurobiological and neuropharmacological research conducted in this area (41). Neuronal antibodies have been primarily identified in rapidly progressive dementia; patients with antibody-mediated dementia may experience significant improvement with immunotherapy, underscoring the vital role of autoantibody discovery in managing cognitive impairment (17, 42). Additionally, circulating autoimmune antibodies have been studied as potential markers of underlying pathological mechanisms of neurodegenerative dementia; most studies have focused on antibodies specific for AD-associated proteins such as Aβ, tau protein and glial markers (11, 13). Few studies have screened for dementia-associated antibodies using human protein microarrays in dementia and MCI (15, 34, 43). We have undertaken comprehensive autoantibodies profiling in 127 individuals with varying degrees of cognitive dysfunction by utilising protein microarray and quantifying over 1,600 autoantibodies, to identify autoantibody signatures and determine their biological significance in patients with MCI and dementia. Of 534 reactive autoantibodies identified, after extensive plasma screening, we identified 33 and 38 altered autoantibodies in patients with dementia compared to individuals with normal cognitive function and MCI, respectively. Autoantibodies to a further 20 proteins were altered significantly in subjects with MCI compared to cognitively normal subjects. GO enrichment analysis showed that the corresponding proteins of these dysregulated autoantibodies were mainly over-represented in binding or catalytic activity and enriched in multiple pathways related to neurodegeneration, including neurotrophin signaling pathway, long-term potentiation, axon guidance, cholinergic synapse, apoptosis, glycolysis and gluconeogenesis. Five autoantibodies in particular, were dysregulated in dementia and MCI, including CAMK2A, CKS1B, ETS2, MAP4, and NUDT2. CAMK2A (Calcium/Calmodulin-Dependent Protein Kinase II Alpha) plays an essential role in synaptic plasticity and memory formation (44), and is expressed exclusively in glutamatergic neurons (45), especially in the hippocampus (46). Our study revealed that antibodies against CAMK2A were downregulated in patients with dementia and MCI. CAMK2 protein is significantly decreased in the hippocampi of patients with AD and MCI and is colocalised with β-amyloid on senile plaques (47–49). CAMK2 dysregulation appears to be a key contributor to synaptic degeneration, and memory deficits (50), with activation of CAMK2 by β-amyloid associated with impairment in long-term potentiation and dendritic spine loss (51, 52). We also show that Anti-MAP4 antibodies were upregulated in patients with MCI and dementia. Autoantibodies against this protein have been reported to be elevated in AD using the same assay (53). MAP4 is a microtubule-associated protein belonging to the MAP2/Tau family that promotes microtubule assembly. Whilst MAP4 exhibits low expression in the nervous system during neuronal development, it is up-regulated in neurons and glia during adulthood (54). MAP4, along with tau and MAP1, have been reported to be homocysteinylated with accumulation in protein aggregates in the brains of patients with AD and vascular dementia (55). In experimental models, this has been suggested to lead to an impaired functional interaction between MAPs and their partner proteins which affects synaptic plasticity and leads to cognitive dysfunction (55). Whilst, CKS1B (CDC28 protein kinase regulatory subunit 1B) plays a role in cell cycle regulation and has been found to be dysregulated in several types of cancer (56), there is limited research on its potential involvement in dementia. However, altered expression of several cell cycle regulatory proteins, including CKS1B, has been reported in the brain of patients with different stages of AD (57, 58). There is limited research on the role of NUDT2 and EST2 in dementia. NUDT2 is a protein that belongs to the nudix hydrolase family and is involved in the regulation of nucleotide metabolism, mutation in the NUDT2 gene was reported to be associated with neurodevelopmental delay and intellectual disability, and polyneuropathies with demyelinating and/or axonal features (59, 60). ETS2 is a transcription factor shown to play a role in cell proliferation, differentiation, and apoptosis (61). ETS2 is a transcriptional regulator of the β-amyloid precursor protein, which is centrally involved in senile plaque formation in Down syndrome and AD (62).

The present study identified dysregulated autoantibodies against proteins involved in several neuro-related pathways that may play a role in the development of dementia. Indeed, our GO analysis showed that RHOA, CAMK2A, RPS6KA6, and NRAS are involved in the neurotrophin signaling pathway, long-term potentiation, and axon guidance. RHOA is a small GTPase of the Rho family. Rho GTPases are essential in regulating neuronal morphology, such as dendritic arborisation, spine morphogenesis, and axon guidance (63) as well as; also, Rho GTPases are key regulators of hippocampal long-term potentiation and depression, the most common forms of synaptic plasticity that are important for learning and memory (64). RhoA signaling is suggested to be involved in several key aspects of AD pathogenesis, including β-amyloid aggregation, tau phosphorylation, neuroinflammation, and synaptic damage (65). RPS6KA6 (RSK4) ribosomal S6-kinase 4 is a member of a family of serine–threonine kinases involved in the MAPK pathway that regulate cell proliferation, survival, growth, and movement (66). RPS6KA6 is most abundantly expressed in the brain and kidney, and deletions of this protein have been reported to be associated with X-linked intellectual disability (67). Interestingly, IgG against RPS6KA6 has been shown to be the most frequently selected autoantibody for predicting ageing and age-related neurodegenerative diseases such as AD and advanced-stage Parkinson’s disease (68). NRAS (N-Ras) protein is an intrinsic GTPase associated with the progression of glioblastoma multiforme and melanoma, by regulating cell migration, growth, and angiogenesis (69–72). It has also been associated with neurodevelopmental disorders through its involvement in the MAPK signaling pathway that plays a critical role in brain function, including learning, memory and synaptic plasticity (73). Whilst there is limited evidence for the involvement of this protein in cognitive dysfunction in adults; a previous study reported high enrichment of NRAS in the brains of a mouse model of AD (74). The neurotrophin signaling pathway was one of the most significantly enriched KEGG pathways in dementia, as shown by our GO analysis. Neurotrophins are growth factors that regulate neuronal development, differentiation, and survival in the central and peripheral nervous system (75) and play a critical role in neuronal survival, neuroplasticity, neurogenesis, and neurovascular integration (76–79). Dysregulation of neurotrophic factor signaling resulting in a lack of trophic support for certain populations of neurons is believed to contribute to the onset and progression of neurodegeneration and cognitive decline (80–83). We propose that the dysregulation of the identified pathways could potentially be associated with the production and class switching of antibodies that target these proteins. Since the regulatory mechanism for these antibodies remains unclear, further investigation is required to understand the basis for the change in antibodies that target these proteins and their potential role in dementia.

Our study has some limitations that should be noted, including the small sample size, which may limit the generalizability of our findings. As a result, it is important to verify the identified autoantibodies in larger, diverse, well-matched, and independent cohorts to elucidate their role in the pathogenesis of dementia. Another limitation lies in our inability to accurately diagnose MCI and dementia based on their underlying etiology. While our study employs the International Classification of Diseases, Tenth Revision (ICD-10) criteria for diagnosing dementia and MCI cases and relies on MRI for categorizing dementia into AD or VD, it’s important to note that current best practices emphasize the use of biomarkers such as amyloid and tau positivity in cerebrospinal fluid or amyloid PET scans for a more accurate diagnosis of AD. Future research should consider incorporating cerebrospinal fluid analysis and PET imaging to establish more precise classifications of specific neurodegenerative conditions. Such an approach would not only enhance our ability to differentiate between different types of dementia but also validate the findings of our current study.

## 5 Conclusion

This study systematically explored the plasma autoimmune profile in dementia and MCI using KREX technology immunoassay consisting of more than 1,600 human proteins. Differential expression analysis revealed 33 and 38 dysregulated plasma autoantibodies in dementia compared to those with normal cognitive function and MCI subjects, and 20 autoantibodies were differentially expressed in MCI. Our study sheds light on potential underlying mechanistic pathways for dementia, as several autoantibodies were reactive against proteins involved in the neurotrophin signaling pathway, long-term potentiation, axonal guidance, the cholinergic synapse, apoptosis, glycolysis and gluconeogenesis. Moreover, the correlation of plasma autoantibodies with cognitive dysfunction revealed a group of potential markers related to disease progression and severity. The significance of these autoimmune changes in subjects with MCI and dementia requires further validation.

## Data availability statement

The original contributions presented in the study are included in the article/Supplementary material, further inquiries can be directed to the corresponding author.

## Ethics statement

The studies involving humans were conducted in accordance with the Declaration of Helsinki and approved by the Institutional Review Board of Qatar Biomedical Research Institute (2019-013), Hamad Medical Corporation (RP14494/14), and Weill Cornell Medicine-Qatar (15-00019), Doha, Qatar. The studies were conducted in accordance with the local legislation and institutional requirements. Written informed consent was obtained from all participants involved in the study.

## Author contributions

HE: Data curation, Formal Analysis, Investigation, Methodology, Visualization, Writing – original draft. AM: Data curation, Formal Analysis. GP: Methodology, Writing – review & editing. KL: Formal Analysis. HH: Resources. MC: Methodology. AP: Methodology. HA: Project administration, Resources, PW: Methodology. JD: Resources. NA: Resources. MR: Methodology. SK: Methodology. RA: Methodology. AO: Methodology. AE: Methodology. OA: Resources. AA: Resources, Writing – review & editing. JB: Resources. RM: Resources, Writing – review & editing. O.M.E-A., Conceptualisation, Supervision, Writing – review and editing, Resources. All authors have read and agreed to the published version of the manuscript.

## Footnotes

^1^ http://bioinformatics.sdstate.edu/go/
