# Supplementary material for: Profiling the autoantibody repertoire reveals autoantibodies associated with mild cognitive impairment and dementia
Source: Front Neurol. 2023 Nov 30;14:1256745. doi: 10.3389/fneur.2023.1256745 (PMC10722091; doi:10.3389/fneur.2023.1256745)
Supplement: Supplementary file 1 [file Data_Sheet_1.ZIP › Supplementry materials/Supplementary_Material.docx]

Supplementary Material

# Supplementary Figures


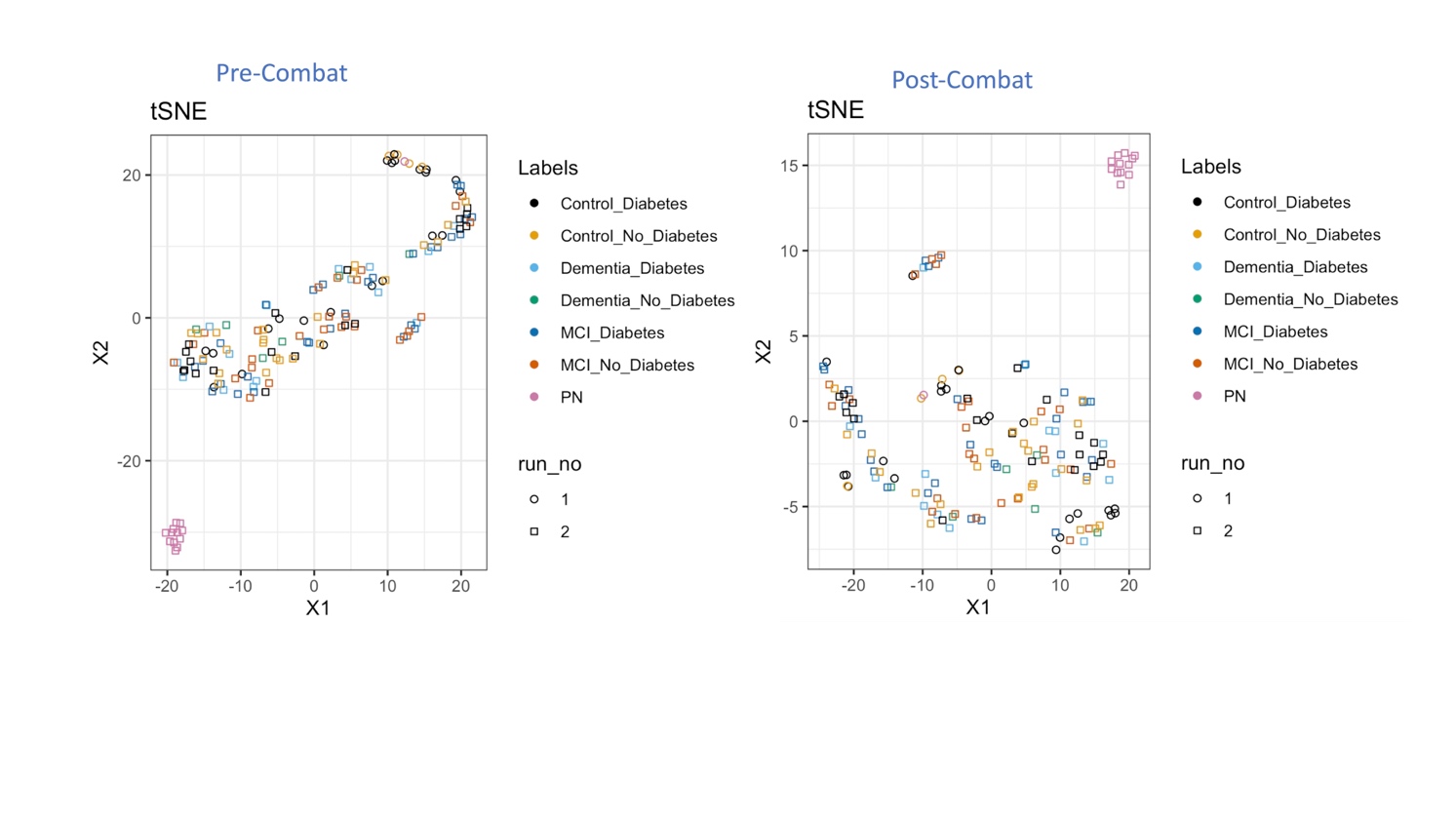
**Supplementary Figure S1.** tSNE exploratory visualisation (on Loess normalised, pre-and post-Combat, NCF dataset). The batch effect was improved by ComBat with no discernable difference by run number for the clinical samples.


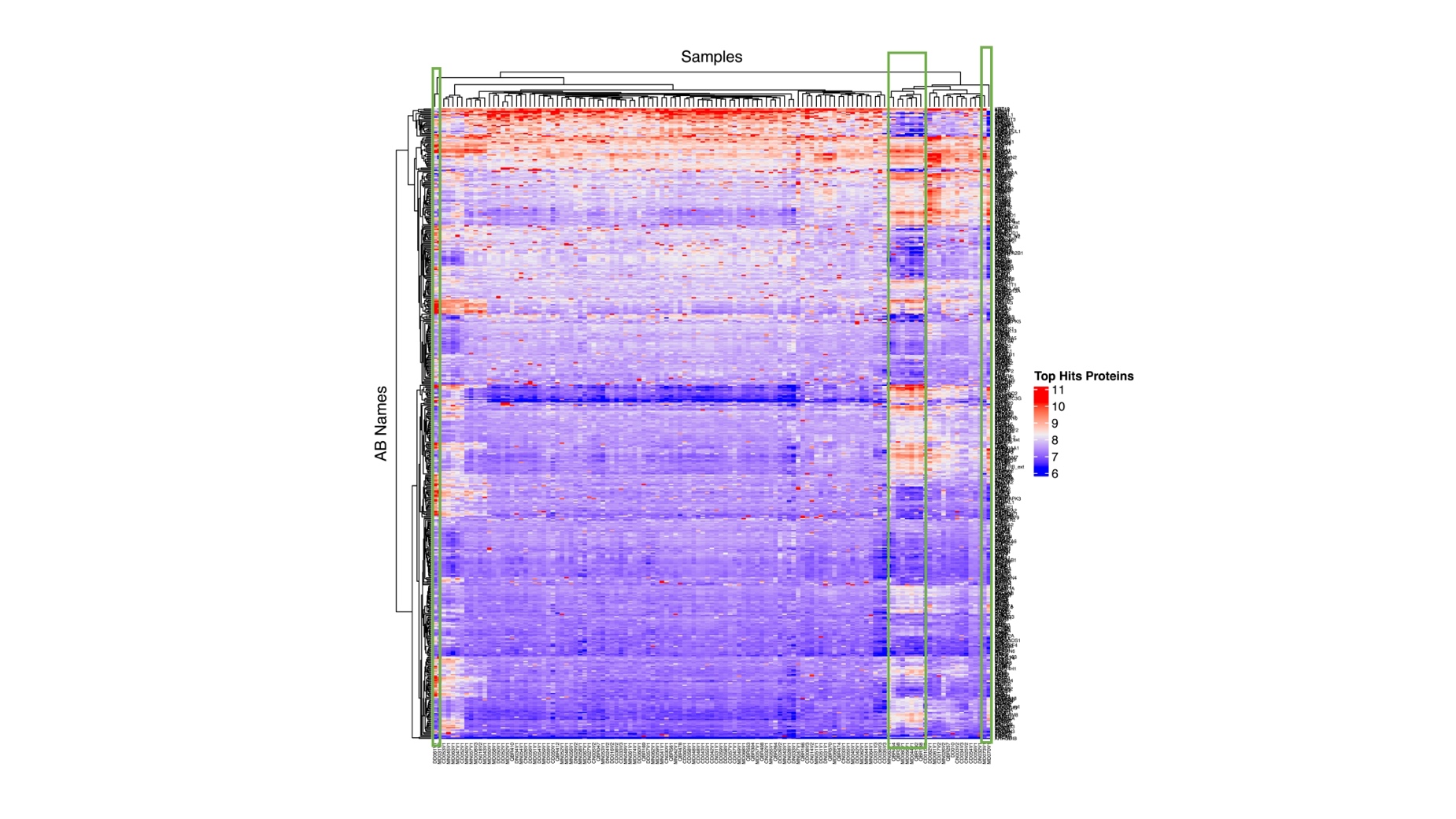


**Supplementary Figure S2.** PSA sample identification (post-loess normalisation).

# Supplementary Tables

**Supplemental Table S1.** Differential expression analysis of the autoantibodies in dementia vs cognitively normal controls identified by limma.

**Supplemental Table S2**. Differential expression analysis of the autoantibodies in dementia vs MCI identified by limma.

**Supplemental Table S3.** Differential expression analysis of the autoantibodies in MCI vs cognitively normal subjects identified by limma.

**Supplemental Table S4.** Correlation of plasma level of autoantibodies with the cognitive performance score (MoCA) in subjects with MCI and dementia.
